# Alcohol-Intoxicated Patients With Blunt Trauma and Head Injuries Have Better Outcomes Than Sober Patients

**DOI:** 10.7759/cureus.63044

**Published:** 2024-06-24

**Authors:** Kazuhiko Takagi, Daizoh Saitoh, Koshi Nakagawa, Hironori Inoue, Hidekazu Takeuchi, Hiroshi Takyu, Hideharu Tanaka

**Affiliations:** 1 Department of Emergency Medical System, Graduate School, Kokushikan University, Tokyo, JPN; 2 Department of Paramedicine, Shinjuku Fire Station, Tokyo Fire Department, Tokyo, JPN; 3 Department of Integrated Science and Engineering for Sustainable Societies, Faculty of Science and Engineering, Chuo University, Tokyo, JPN

**Keywords:** nationwide trauma registry, glasgow outcome scale, survival, drinking alcohol, head injury

## Abstract

Background: Although alcohol-intoxicated patients have difficulties evaluating their consciousness level and being transported prehospital, there is some evidence that the survival outcomes for alcohol-intoxicated patients with head injuries are better. The present study evaluated whether the survival and brain function outcomes in alcohol-intoxicated trauma patients with head injuries were better than those in sober patients using the Japan Trauma Data Bank (JTDB), a nationwide trauma registry in Japan.

Methods: The 17,823 patients with blunt trauma, including head injuries, who were registered in the JTDB database between January 2019 and December 2021 were retrospectively analyzed. Logistic regression analyses were performed for in-hospital survival in patients with blunt trauma, including those with head injuries, and for good brain function based on the Glasgow outcome scale (GOS) in patients with only head injuries. Survival rates by head injury score using the abbreviated injury scale (AIS) 2008 or injury severity score (ISS) categories were compared between drinking and nondrinking groups.

Results: Drinking significantly affected survival (odds ratio 1.800, p<0.001) and good brain function (odds ratio 1.546, p<0.001), as indicated by logistic regression analysis using head injuries alone or blunt multisite trauma (including head injuries), respectively. According to analyses by the ISS category or head AIS score, there were significant differences between the drinking and non-drinking groups in several categories (ISS 9-15, 16-24, and 25-40 and AIS 3 and 5) regarding survival rates with blunt trauma, including head injuries, or good GOS rates with head injuries alone.

Conclusions: The survival rates for blunt trauma, including head injuries, and the prognosis for brain function based on the GOS were better in the drinking group than in the control group for cases with head injuries alone. A multivariate analysis also showed that alcohol consumption was significantly associated with better outcomes.

## Introduction

Alcohol consumption increases the risk of injury [1,2]. Additionally, emergency paramedics face difficulties in evaluating the consciousness level of alcohol-intoxicated patients [3] and transporting them in the prehospital field. However, the survival of alcohol-intoxicated patients with head injuries is considered to be comparatively better than that of non-drinking head injury patients [3-7].

In a 2006 study of 1,158 patients with head injuries transported to a trauma center in Toronto, Canada, Tien et al. reported that patients with low-to-moderate blood alcohol levels had a better life expectancy than those who had not been drinking [4]. Scheenen et al. further reported that in cases of mild traumatic brain injury, the drinking group had a better prognosis regarding brain function at six months compared to the non-drinking group [3]. Salim et al. reported a significantly lower mortality rate in patients in whom alcohol drinking was detected than in non-drinking patients in their study of 482 patients with head injuries [5]. Additionally, Raj et al. reported better outcomes in patients with traumatic brain injuries and higher blood alcohol levels than in others [6]. However, Nojima et al. [8] found that pre-injury alcohol consumption did not improve neurological outcomes in patients with severe traumatic brain injury in their study using the Japanese Trauma Data Bank (JTDB). Therefore, it is unclear whether alcohol consumption before traumatic brain injury is protective or not, indicating caution on this matter.

To date, academic studies on alcohol consumption and trauma have primarily focused on isolated cases of head injuries. It can be challenging to ascertain in the prehospital setting whether blunt trauma patients who have consumed alcohol have sustained a solitary head injury. Addressing this issue statistically could greatly benefit prehospital care for alcohol-intoxicated patients with multisite trauma. However, to our knowledge, no previous studies have demonstrated a superior prognosis among alcohol-consuming patients with blunt, multisite trauma compared to sober patients.

The present study aimed to assess whether survival and brain function outcomes in alcohol-consuming patients with blunt trauma, including head injuries, were superior to those in non-drinking patients, using the JTDB [9-12], a prominent nationwide trauma registry in Japan.

## Materials and methods

Study design

The Medical Ethics Committee of the Graduate School of Emergency Medicine of Kokushikan University in Tokyo, Japan, approved the use of data from the JTDB, a nationwide trauma registry, for this retrospective cohort study (approval number: 23016). Participation in JTDB data collection began in 55 hospitals in January 2004. The number of participating hospitals has increased annually, and as of April 2022, 303 hospitals have participated in JTDB data collection. Notably, since 2019, the JTDB has transitioned from its previous database system to the current one. We adhered to JTDB rules and utilized data from the new system for our study.

The JTDB collects prehospital and in-hospital information, including patient demographics, injury type, means of transportation, vital signs, in-hospital procedures, abbreviated injury scale (AIS) 2005 update 2008 [13], Glasgow outcome scale (GOS) [14], and in-hospital mortality. The need for informed consent was waived due to the anonymity and retrospective nature of the study.

Patient selection

The patients included in the study were those with acute blunt trauma, excluding those who experienced cardiopulmonary arrest upon arrival. We also excluded patients without signs of life upon hospital arrival, those without head injuries, and those with an unknown drinking habit. The present analysis included all patients admitted to hospitals registered in the JTDB database between January 2019 and December 2021 who suffered from blunt trauma.

Data collection

Among the items available in the JTDB (2019-2021; new system), the following variables were used in this study: age, sex, type of trauma, systolic blood pressure (sBP) at hospital arrival, GCS at the prehospital site and hospital arrival [15], revised trauma score (RTS) at hospital arrival [16], injury severity score (ISS) [17], maximum score in body region 1 of the AIS 2005 update 2008 (AIS 2008) [13], in-hospital survival, and GOS [14]. To establish a group of patients with only head injuries and no trauma to other regions, we selected patients using the AIS 2008 maximum score in body region 1. The number of patients with only head injuries and no other trauma was 7,370. The outcomes assessed were in-hospital survival and GOS.

Statistical analyses

Categorical variables are presented as numbers and percentages, while quantitative variables are presented as means and standard deviations (or standard errors). The chi-squared test was used to compare categorical variables, and Welch’s t-test was used to compare quantitative variables. Changes in the level of consciousness over time were tested with a repeated measures ANOVA. Statistical significance was set at p<0.05

Multivariable logistic regression analyses were performed to examine the relationship between in-hospital survival and variables in cases involving multisite injuries. For cases involving multisite injuries, the criterion was in-hospital survival, and the explanatory variables were age, sex, ISS, RTS, GCS score on hospital arrival, AIS maximum score in region 1 (head region), and alcohol consumption. Additionally, another logistic regression analysis was conducted to examine the relationships between good outcomes based on the GOS (good outcome 1, GOS 4 and 5; poor outcome 0, GOS 1-3) and variables for head injuries alone. The criterion variable was a good GOS, and the explanatory variables were age, sex, RTS, AIS maximum score in region 1, and alcohol consumption. Odds ratios, p-values, and corresponding 95% confidence intervals (CIs) are reported.

The survival rates according to the ISS categories or the good GOS rates by head injury grade according to the AIS 2008 maximum score in region 1 were compared using the chi-squared test between the drinking and control groups, involving cases of multisite injuries or head injuries alone. Statistical analyses were performed using SPSS Statistics version 22 (IBM Corp., Released 2013, IBM SPSS Statistics for Windows, Version 22.0. Armonk, NY: IBM Corp.).

## Results

**Figure 1:**
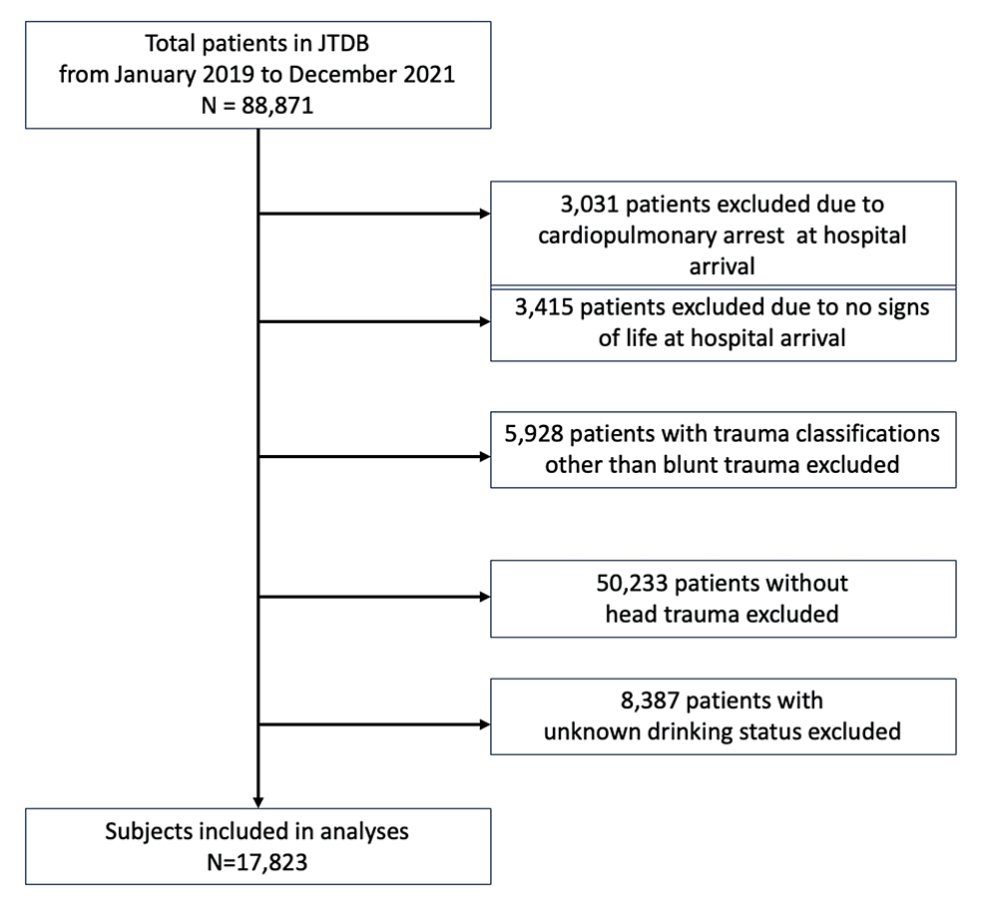
Study flow diagram of patients included A total of 88,871 patients registered in the JTDB new database, published in 2022, were identified. After exclusion, 17,823 patients met the inclusion criteria. JTDB: Japan Trauma Data Bank

A patient collection diagram is shown in Figure 1. We identified 88,871 patients with blunt trauma registered in the JTDB database published in 2022. After exclusions, a total of 17,823 patients met the inclusion criteria.

The profiles of the patients in the drinking (n=2,622) and non-drinking (n=15,201) groups are shown in Table 1. The mean age was 56.5 years old in the drinking group and 61.1 years old in the control group. Men were more frequent than women in both groups, with male patients constituting 84.2% (N=2,205) in the drinking group and 63.7% (N=15,122) in the control group, respectively. The mean ISS was 15.1 and 16.2, the mean RTS was 7.10 and 7.28, the mean systolic BP was 135 and 144, the mean prehospital GCS was 11.1 and 12.4, the mean GCS at hospital arrival was 11.9 and 12.6, and the mean AIS maximum score in region 1 was 3.0 and 2.9 in the drinking and control groups, respectively. All elements, except for the AIS maximum score in region 1, differed significantly between the two groups due to the large sample size.

**Table 1:** Profile of the drinking and control groups Data for items other than gender were shown as mean ± standard deviation. Statistical significance was set at p<0.05. ISS: injury severity score, RTS: revised trauma score at arrival of hospital, AIS: abbreviated injury scale 2005 update 2008, sBP: systolic blood pressure at hospital arrival, GCS: Glasgow coma scale, AIS region 1 max: maximum score of AIS head region in the case

	Drinking group (N=2,622)	Control group (N=15,201)	p-value
Age	56.5±18.1 (2,619)	61.1±26.2 (5,184)	<0.001
Gender (male)	84.2% (2,605)	63.7% (15,122)	<0.001
ISS	15.1±9.5 (2,612)	16.2±10.3 (15,156)	<0.001
RTS	7.10±1.20 (2,411)	7.28±1.08 (14,002)	<0.001
sBP	135±30 (2,600)	144±34 (14,954)	<0.001
GCS at the prehospital site	11.1±4.6 (1,192)	12.4±3.9 (7,834)	<0.001
GCS at hospital arrival	11.9±3.8 (2,520)	12.6±3.5 (14,812)	<0.001
AIS region 1 max	3.0±1.2 (2,622)	2.9±1.2 (15,201)	0.295

**Figure 2:**
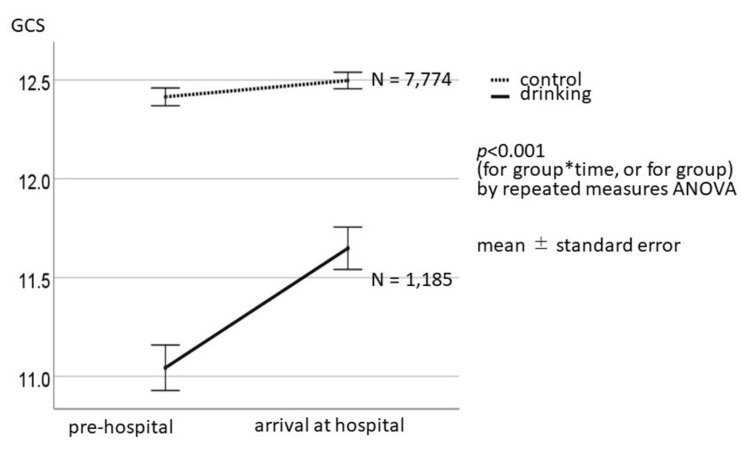
Changes in the total GCS between the two groups between the injury site and hospital arrival The p-value was <0.001 from a repeated measures analysis of variance with respect to the changes in the GCS between the injury site and hospital arrival. Statistical significance was set at p<0.05. GCS: Glasgow coma scale, ANOVA: analysis of variance

The changes in the total GCS score between the two groups from the injury site to hospital arrival are shown in Figure 2. The GCS score in the drinking group significantly changed (elevated) from the injury site to hospital arrival in comparison to that in the non-drinking group.

**Figure 3:**
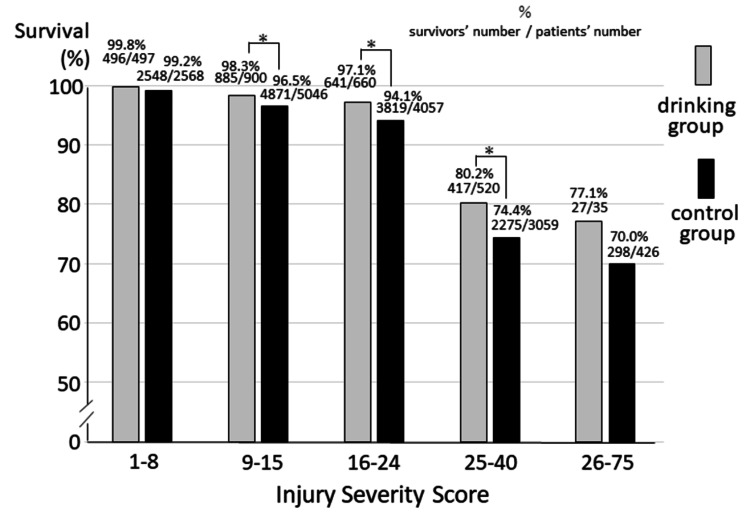
Comparison of the survival rates between the two groups by ISS category using multisite trauma patients, including those with head injuries Survival rates for patients with ISS 9-15, 16-24, or 25-40 were significantly different between the drinking and control groups (ISS 9-15: p=0.005, ISS 16-24: p=0.002, ISS 25-40: p=0.004 by chi-square test; statistical significance was set at p<0.05). * p<0.05, ISS: injury severity score, % survivors’ number was divided by patients’ number

We compared survival between the two groups based on ISS categories using multisite trauma patients, including those with head injuries (Figure 3). For patients in ISS categories 9-15, 16-24, and 25-40, those in the drinking group had significantly higher survival rates at discharge than those in the control group (chi-squared test). Overall, 2,474 (94.4%) out of 2,622 patients survived in the drinking group, whereas 13,845 (91.1%) out of 15,201 patients survived in the non-drinking group. The survival outcome of patients with blunt trauma who were drinking, including those with head injuries, was also better than that of non-drinking trauma patients.

The results of the logistic regression analysis on the survival rate of patients with trauma, including those with head injuries, are shown in Table 2. Drinking was found to be a factor associated with better survival. Interestingly, the survival rate among blunt trauma patients, including those with head injuries, was higher in the drinking group than in the control group. Table 2 presents the odds ratios, p-values, and 95% CIs, with age, sex, ISS, RTS on hospital arrival, GCS on hospital arrival, and AIS maximum score in region 1 used as covariates.

**Table 2:** Survival rates of blunt trauma patients, including those with head injuries, using a logistic regression analysis ISS: injury severity score, RTS: revised trauma score at hospital arrival, GCS: Glasgow coma scale at hospital arrival, AIS: abbreviated injury scale 2005 update 2008, AIS region 1 max: maximum score of AIS head region in the case, CI: confidence interval

	Odds ratio	p-value	95% CI
Age	0.958	<0.001	0.954-0.962
Gender (female)	1.521	<0.001	1.308-1.769
ISS	0.979	<0.001	0.971-0.987
RTS	1.565	<0.001	1.369-1.788
GCS at hospital arrival	1.152	<0.001	1.100-1.205
AIS region 1 max	0.613	<0.001	0.565-0.664
Drinking	1.8	<0.001	1.434-2.261

**Figure 4:**
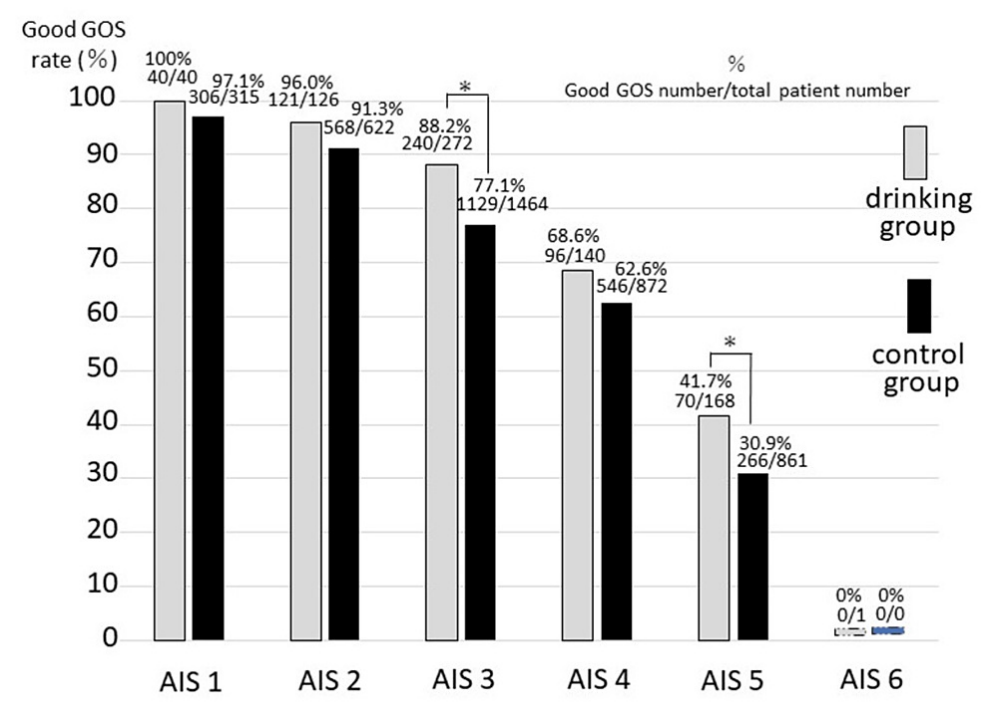
Comparison of the good GOS rates between the two groups by head AIS maximum score category, using patients with head injuries alone Good GOS rates for cases with a maximum AIS of 3 or 5 were significantly different between the drinking and control groups (AIS 3, p=0.012; AIS 5, p=0.009 by chi-square test; statistical significance was set at p<0.05). ＊p<0.05, GOS: Glasgow outcome scale, good GOS rate: the ratio of scores 4 and 5 among all scores, % survivors’ number was divided by patients’ number

A comparison of the good GOS rate between the two groups by the AIS maximum score in region 1 in patients with head injuries alone is shown in Figure 4. Good GOS rates (ratio of scores 4 and 5 among all scores) among patients with a maximum AIS of 3 or 5 differed significantly between the drinking and control groups (chi-squared test). Overall, 567 of 747 patients (75.9%) in the drinking group had good brain function, and 2,815 of 4,134 patients (68.1%) in the non-drinking group had good brain function.

The results of the logistic regression analysis of the good GOS rate among patients with head injuries alone are shown in Table 3. In this multivariate analysis, alcohol consumption was found to significantly (p<0.001) influence the GOS rate.

**Table 3:** Good GOS rates of patients with head injuries alone using a logistic regression analysis RTS: revised trauma score at hospital arrival, AIS: abbreviated injury scale 2005 update 2008, AIS region 1 max: maximum score of AIS head region in the case, CI: confidence interval, CPC: cerebral performance category, GOS: Glasgow outcome scale

	Odds ratio	p-value	95% CI
Age	0.953	<0.001	0.948-0.958
Gender (female)	1.423	<0.001	1.196-1.692
RTS	2.289	<0.001	2.099-2.496
AIS region 1 max	0.474	<0.001	0.435-0.517
Drinking	1.546	<0.001	1.203-1.987

## Discussion

A new finding of this study is that the survival rate of blunt trauma patients with head injuries, including multisite trauma patients, was significantly better in the drinking group than in the non-drinking group (Figure 3 and Table 2). In the field, it is often difficult to determine whether a patient has experienced only head injuries or trauma in areas other than the head. Blunt trauma cases are more likely to involve trauma to other parts of the body than head injuries. Therefore, the results of the present study demonstrate realistic value for pre-hospital field paramedics. Furthermore, the consciousness of alcohol-intoxicated patients with blunt trauma, including head injuries, differed at the scene and upon arrival at the hospital (Figure 2). Alcohol-intoxicated patients often exhibit resistance to prehospital activities, posing challenges for emergency paramedics. However, encouraging paramedics and emergency physicians to consider that such patients may already have characteristics conducive to a positive outcome could contribute to a paradigm shift in rescue care concepts. Therefore, the results of this study, utilizing data from the nationwide JTDB, hold significant social implications.

The results of the present analysis of cases of head injuries alone show that alcohol consumption has an unequivocally favorable impact on brain function prognosis at discharge from the hospital. The AIS 2008 maximum score for body region 1 can be considered a categorization of the severity of head injuries. The results of the GOS-based analysis of brain function prognosis (Figure 4 and Table 3) showed an improvement in alcohol-intoxicated patients with head injuries alone. In cases with head injuries alone, the drinking group had significantly better results than the non-drinking group regarding in-hospital survival using the same method (data not shown). The database used in this study, JTDB (published in 2022, comprising cases from 2019 to 2021), covers severe trauma cases in Japan. Therefore, we believe that this study allowed us to verify that patients who have been drinking alcohol with head injuries alone are associated with better outcomes than similar patients who have not been drinking alcohol.

It is not yet clear why alcohol consumption increases survival rates and improves brain function prognosis after head injuries and other injuries. However, various mechanisms have been proposed for this effect of alcohol. One report suggests that alcohol inhibits the effects of the surge in catecholamines that occurs with severe head injuries [18]. After severe head injuries, catecholamines released from large quantities of destroyed neurons cause over-excitation of the sympathetic nervous system, which in turn triggers various organ failures accompanied by inflammation. NMDA-type glutamate receptors are said to play a role in this process, and alcohol blocks these receptors, breaking the adverse chain reaction [6].

Additionally, some studies have shown that alcohol increases corticosterone, which exerts an anti-inflammatory effect in the brains of rats, or that alcohol drinking is associated with fewer complications of pneumonia [19]. Therefore, alcohol might be key to improving the outcomes of trauma patients.

Limitations

Several limitations of the present study warrant mention. First, as this is a retrospective study using big data from Japan, the findings from this study in a monoethnic Japanese population may not be applicable worldwide. Second, the JTDB does not provide the blood alcohol concentration of patients after they arrive at the hospital, even though the blood alcohol concentration is measured at each facility and data is entered. Therefore, since the blood alcohol concentration of each case was not known, cases with a characteristic odor due to drunkenness and whose drinking history was learned after arrival at the hospital were treated as “drinking cases” in this study. Third, the results of this study are based on the accreditation results of 303 registered hospitals; therefore, the level of trauma care may not be consistent among hospitals, which can affect mortality rates and brain function prognoses.

## Conclusions

This study compared the in-hospital survival rate and brain function outcomes of blunt trauma patients between the drinking and non-drinking groups using the JTDB. The survival rates for blunt trauma, including head injuries, and the prognosis for brain function based on the GOS in cases with head injuries alone were better in the drinking group than in the control group. The multivariate analysis also showed that alcohol consumption was significantly associated with better outcomes.
